# *Ty‐1*, a universal resistance gene against geminiviruses that is compromised by co‐replication of a betasatellite

**DOI:** 10.1111/mpp.12885

**Published:** 2019-11-22

**Authors:** Corien M. Voorburg, Zhe Yan, Maria Bergua‐Vidal, Anne‐Marie A. Wolters, Yuling Bai, Richard Kormelink

**Affiliations:** ^1^ Laboratory of Virology Wageningen University & Research Droevendaalsesteeg 1 Wageningen 6708PB Netherlands; ^2^ Plant Breeding Wageningen University & Research Droevendaalsesteeg 1 Wageningen 6708PB Netherlands

**Keywords:** beet curly top virus, betasatellite, geminivirus, resistance, RNA interference, tomato yellow leaf curl virus, *Ty‐1*

## Abstract

Tomato yellow leaf curl virus (TYLCV), a begomovirus, causes large yield losses and breeding for resistance is an effective way to combat this viral disease. The resistance gene *Ty‐1* codes for an RNA‐dependent RNA polymerase and has recently been shown to enhance transcriptional gene silencing of TYLCV. Whereas *Ty‐1* was earlier shown to also confer resistance to a bipartite begomovirus, here it is shown that *Ty‐1* is probably generic to all geminiviruses. A tomato *Ty‐1* introgression line, but also stable transformants of susceptible tomato cv. Moneymaker and *Nicotiana benthamiana (N. benthamiana)* expressing the *Ty‐1* gene, exhibited resistance to begomoviruses as well as to the distinct, leafhopper‐transmitted beet curly top virus, a curtovirus. Stable *Ty‐1* transformants of *N. benthamiana* and tomato showed fewer symptoms and reduced viral titres on infection compared to wild‐type plants. TYLCV infections in wild‐type *N. benthamiana* plants in the additional presence of a betasatellite led to increased symptom severity and a consistent, slightly lowered virus titre relative to the high averaged levels seen in the absence of the betasatellite. On the contrary, in *Ty‐1* transformed *N. benthamiana* viral titres increased in the presence of the betasatellite. The same was observed when these *Ty‐1*‐encoding plants were challenged with TYLCV and a potato virus X construct expressing the RNA interference suppressor protein βC1 encoded by the betasatellite. The resistance spectrum of *Ty‐1* and the durability of the resistance are discussed in light of antiviral RNA interference and viral counter defence strategies.

## Introduction

Tomato yellow leaf curl virus (TYLCV) is the representative of the Old World monopartite begomoviruses within the family *Geminiviridae*. The virus belongs to the most devastating plant viruses worldwide and causes major losses in economically important crops like tomato. The virus mainly occurs in the (sub)tropical and Mediterranean regions worldwide due to the distribution of its vector, the whitefly *Bemisia tabaci (B. tabaci)* (Moriones and Navas‐Castillo, 2000). TYLCV, and all monopartite begomoviruses, have a single, circular, single‐stranded DNA genome of *c*. 2.7 kb in size. In the field, monopartite begomoviruses are frequently observed with co‐replicating alpha‐ or betasatellites (Nawaz‐ul‐Rehman and Fauquet, 2009). Both satellites are approximately half the size of begomovirus genomes. While less is known about alphasatellites and their role in the pathogenesis of begomoviruses, co‐replication of betasatellites often leads to more severe disease symptoms (Zhou, 2013); therefore, betasatellites are regarded as pathogenicity determinants. Betasatellites code for one single protein, βC1, that is known to suppress the antiviral defence mechanism transcriptional gene silencing (TGS) (Yang *et al.*, 2011; Zhou, 2013).

Because the vector *B. tabaci* is difficult to control, the most effective way to combat TYLCV infections is to breed for resistance. Six resistance genes are known, namely *Ty‐1* to *Ty‐6*, and some of these are widely used for introgression breeding. Five of these genes are derived from wild tomato species: *Ty‐1*, *Ty‐3*, *Ty‐4* and *Ty‐6* are from *Solanum chilense (S. chilense), Ty‐2* is from *S. habrochaites,* while *ty‐5* was identified in an old commercial tomato cultivar Tyking (Hanson *et al.*, 2006; Hutton and Scott, 2014; Hutton *et al.*, 2012; Ji *et al.*, 2007, 2009; Lapidot *et al.*, 2015; Zamir *et al.*, 1994). On viral challenge, plants containing any of these genes do not seem to completely abolish infection as reduced virus titres are still observed in comparison to susceptible cultivars, nor is a hypersensitive response seen (Castro *et al.*, 2005; Maruthi *et al.*, 2003; Picó *et al*., 1999, 2000). In recent years several of these genes have been cloned and characterized; only *Ty‐4* and *Ty‐6* are not. *Ty‐2* encodes a nucleotide‐binding leucine‐rich repeat (NB‐LRR) protein, while the *ty‐5* gene encodes an mRNA surveillance factor and contains a mutation that hampers viral protein translation (Lapidot *et al.*, 2015; Yamaguchi *et al.*, 2018). *Ty‐1* and *Ty‐3* are allelic and code for an RNA‐dependent RNA polymerase (RDR) from the γ‐class and distinct from the well‐established RDRs from the α‐class with recognized functions in the amplification of the antiviral RNA interference (RNAi) response (Verlaan *et al.*, 2013). Until recently, no function was assigned to any of the RDRs from the γ‐class, but with the identification of *Ty‐1* a new class of resistance genes was unveiled.

Besides a major role in gene regulation and chromosome dynamics, RNAi (also named RNA silencing) presents a major antiviral defence mechanism in plants (Shou‐Wei and Voinnet, 2007). The mechanism is induced by double‐stranded (ds) RNA molecules, which arise from viral replicative intermediates or secondary RNA folding structures in genomic or messenger RNA molecules. After their processing by Dicer‐like proteins (DCL) into small interfering (si) RNA molecules of 21–24 nucleotides (nt), one strand of the siRNA is uploaded into an RNA‐induced silencing complex (RISC), leading to its activation (Hammond, 2005). When this complex is loaded with a 21 nt siRNA strand and contains an Argonaute 1 (AGO1) core component, it is able to target RNA molecules with sequence complementarity to the siRNA strand, leading to their degradation or translational arrest (Mallory and Vaucheret, 2010). This process is generally referred to as post‐transcriptional gene silencing (PTGS). In contrast, AGO4 containing RISC is loaded with a 24 nt siRNA strand. This complex targets cytosine methyltransferases to complementary DNA molecules, causing cytosine methylation within the target sequence. This methylation leads to TGS of the DNA sequence (Mallory and Vaucheret, 2010).

The RNAi response in plants, in contrast to insects and animals, is amplified by RDRs (Donaire *et al.*, 2008; Wang *et al.*, 2010). During this process aberrant RNA molecules, resulting from the first cleavage by the slicer activity of AGO1, are recognized by RDRs and converted into more dsRNA molecules of the target sequence. Their processing by DCLs produces a second generation of siRNAs. Due to this amplification a strong (antiviral) RNAi response is mounted. In the *Arabidopsis thaliana* genome, six RDR genes have been annotated, encoding RDR1 to RDR6. From those, RDR1, RDR2 and RDR6 represent the α‐class, whereas RDR3, RDR4 and RDR5 fall into the γ‐class (Willmann *et al.*, 2011). Silencing of RDR1 and RDR6 from the α‐class reduces the antiviral PTGS response and makes plants highly susceptible to RNA viruses, but the RNAi response against geminiviruses does not seem to be affected much by silencing of these RDRs (Aregger *et al.*, 2012; Raja *et al.*, 2008; Yu *et al.*, 2003). This finding not only points to the involvement of other RDRs in the RNAi response against geminiviruses, but also supports the idea that the TGS pathway is the most important RNAi pathway against geminiviruses. In agreement with this hypothesis are findings showing that plants knocked out for proteins from the TGS pathway are hypersusceptible to geminivirus infection (Jackel *et al.*, 2016; Raja *et al.*, 2008). After the identification of *Ty‐1* as an RDR γ‐class member, its involvement in the RNAi amplification was demonstrated by an increase of siRNA production and an elevated rate of cytosine methylation in viral DNA collected from TYLCV‐challenged *Ty‐1* tomato plants (Butterbach *et al.*, 2014).

While the *Ty‐1* resistance gene is generally deployed to combat TYLCV, it has been shown to confer resistance to begomoviruses other than TYLCV (Barbieri *et al.*, 2010; Butterbach *et al.*, 2014; Pietersen and Smith, 2002; Prasanna *et al.*, 2015; Shahid *et al.*, 2013). However, whether the resistance is also effective against members of other genera, e.g. the *Curtovirus* genus, is still unknown. Furthermore, all previous studies on *Ty‐1* so far have been conducted using introgression lines and therefore it still remains to be questioned whether the resistance is solely due to the expression of *Ty‐1* or could involve other chromosomal introgression(s). Lastly, the earlier study by Butterbach *et al. *(2014) pointed towards an Achilles’ heel of *Ty‐1* resistance, namely suppression of TGS by viral RNAi suppressor proteins. In that study the *Ty‐1* resistance against TYLCV was compromised by a co‐infection with cucumber mosaic virus (CMV) (Butterbach *et al.*, 2014). This effect was attributed to the CMV‐encoded RNAi suppressor 2b that was shown to inhibit AGO4 activity during TGS (González *et al.*, 2010; Hamera *et al.*, 2012). Since betasatellites encode suppressors of TGS, the compromising nature of betasatellites, by their encoded βC1 protein, on *Ty‐1* resistance could be anticipated.

Here it is shown that *Ty‐1* from a tomato introgression line, but also after stable transformation into susceptible tomato Moneymaker (MM) and *Nicotiana benthamiana (N. benthamiana)*, confers resistance to a completely distinct leafhopper transmitted curtovirus, beet curly top virus (BCTV). Furthermore, TYLCV resistance in transgenic *Ty‐1* plants is compromised not only by a co‐replicating betasatellite, but also by transient co‐expression of its encoded TGS suppressor protein only.

## Results

### Broad‐spectrum resistance against geminiviruses in *Ty‐1* introgression line

**Figure 1:**
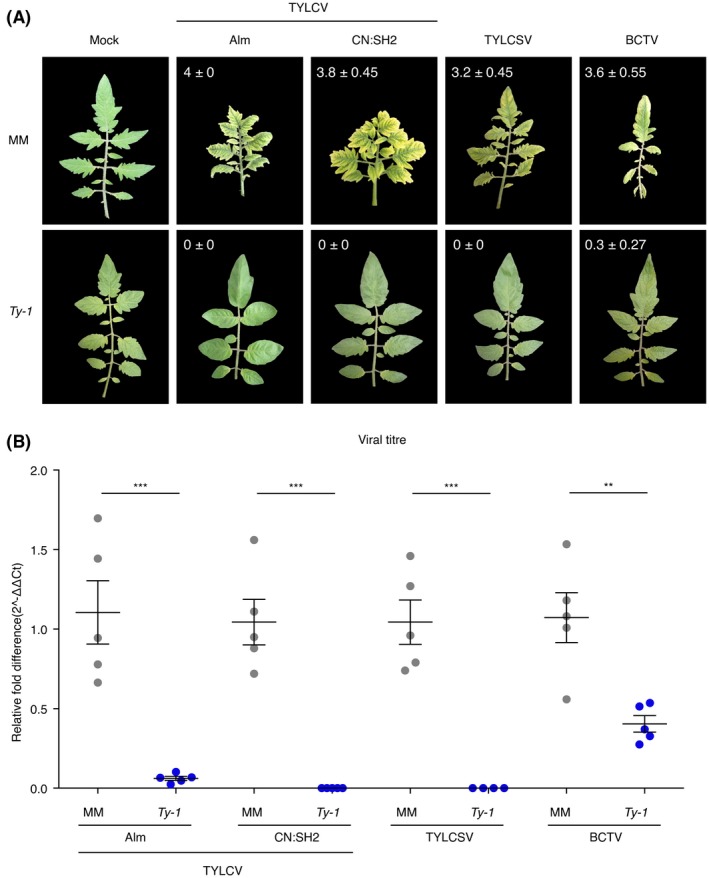
The *Ty‐1* introgression line is resistant against a broad range of geminiviruses. (A) Symptoms upon infection with mock‐inoculation, TYLCV Almeria isolate (Alm), TYLCV‐[CN:SH2], TYLCSV and BCTV in susceptible control (*Solanum lycopersicum* 'Moneymaker', MM) and *Ty‐1* introgression plants at 50 days post‐inoculation (dpi). In the left upper corners the symptom scores are depicted as mean of five biological replicates ± the standard deviation. (B) Virus titre quantification of TYLCV‐Alm, TYLCV‐[CN:SH2], TYLCSV and BCTV in MM and the *Ty‐1* introgression line. Values were normalized relative to tomato *EF1α* and calibrated to the levels in MM plants (set to 1). Dots represent the relative virus titre of individual plants. Lines represent means and standard error of the mean of biological replicates. Asterisks indicate significant differences between *Ty‐1* introgression line and MM according to one‐way analysis of variance (**P < 0.01, ***P < 0.001).

*Ty‐1* has been shown to confer resistance not only to the monopartite TYLCV, but also to the bipartite begomovirus tomato severe rugose virus (ToSRV) (Butterbach *et al.*, 2014). In light of its role in the amplification of RNAi, a mechanism that is antiviral and generic to all viruses, it was anticipated that *Ty‐1* resistance would not be restricted to species from the TYLCV cluster. To test this hypothesis and determine the resistance spectrum of *Ty‐1*, a *Ty‐1* breeding line was challenged with various TYLCV‐like viral species, i.e. TYLCV isolates from Spain and China (both belonging to the TYLCV Israel species), tomato yellow leaf curl Sardinia virus (TYLCSV), as well as a completely distinct geminivirus, namely the leafhopper‐transmitted BCTV, which is a representative of the *Curtovirus* genus. Plants were monitored for 7 weeks by scoring disease symptoms and viral titres were determined. Whereas the susceptible control MM showed severe disease symptoms after a challenge with all (curto‐ and begomo‐) viruses, the *Ty‐1* breeding line remained (almost completely) symptomless on a challenge with the TYLCV Almeria isolate, TYLCV‐[CN:SH2], TYLCSV and BCTV (Fig. 1A). Subsequently, the virus titres were determined via quantitative PCR (qPCR) and the fold difference in titre between the susceptible MM plants and the *Ty‐1* introgression line calculated using the ΔΔ*C*_t_ method (Livak and Schmittgen, 2001). While in MM plants titres were high for all four viruses (*C*_t_ values between 14 and 16), their amounts were significantly reduced in *Ty‐1* introgression lines (Fig. 1B). *Ty‐1* resistance seemed most effective to TYLCSV and TYLCV‐[CN:SH2], for which (hardly) no virus was detected in *Ty‐1* plants (*C*_t_ values between 32 and 34). TYLCV and BCTV were still clearly detected in *Ty‐1* plants (*C*_t_ values between 18 and 20), but TYLCV titres dropped *c*. 18‐fold and BCTV titres *c*. 3‐fold in *Ty‐1* plants compared to MM plants (Fig. 1B).

### Transgenic expression of *Ty‐1* in tomato confers broad‐spectrum resistance to geminiviruses

**Figure 2:**
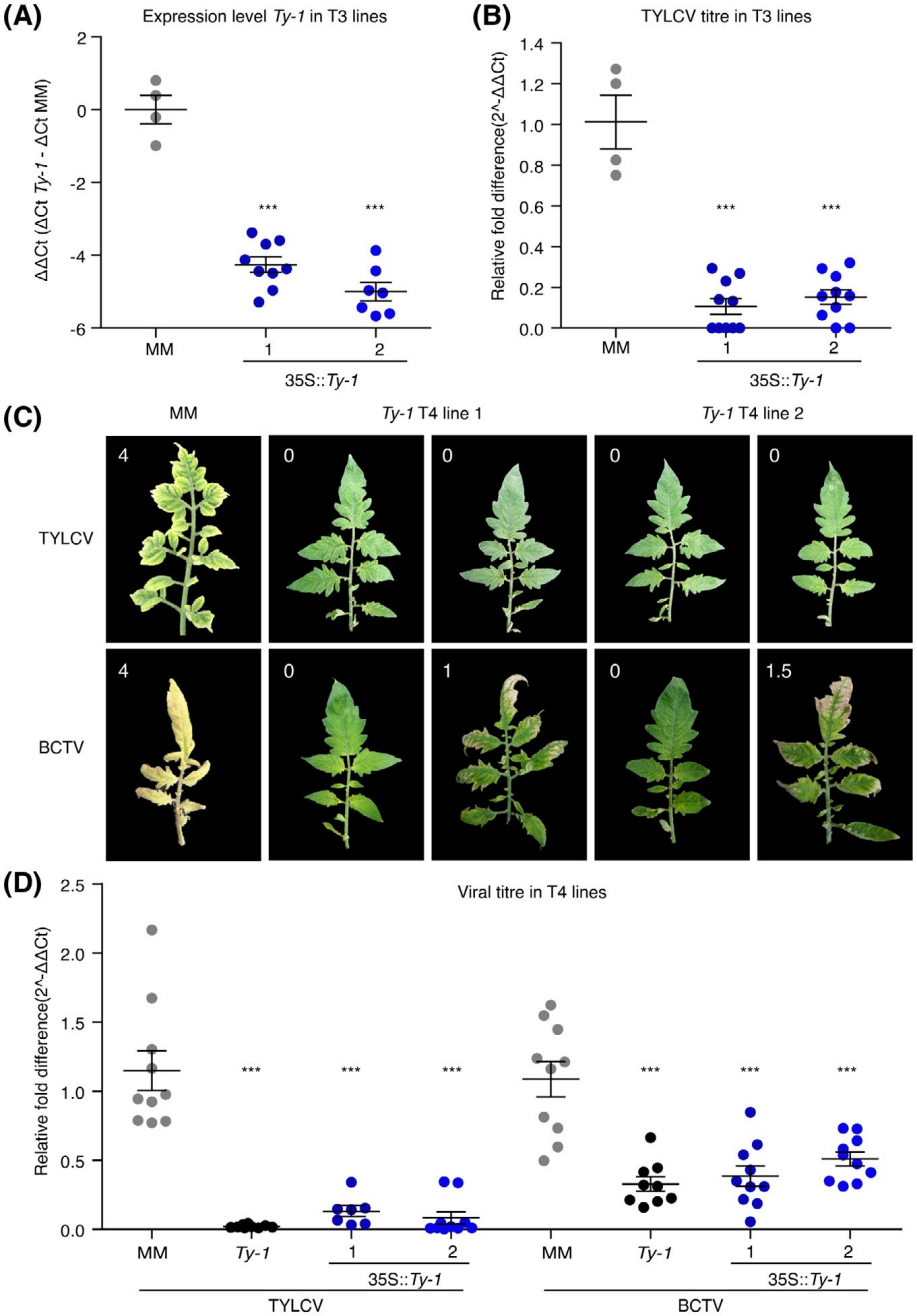
Transgenic tomato plants expressing *Ty‐1* are resistant against both BCTV and TYLCV. For resistant control (*Ty‐1* introgression line) and susceptible control (*Solanum lycopersicum* 'Moneymaker', MM), five plants were included; ten plants of each transgenic line were tested. In all graphs lines represent means and standard error of the mean of biological replicates, dots represent individual plants. (A) Transcript levels of *Ty‐1* in T_3_ families. Values were normalized relative to *EF1α*. The ΔΔ*C*_t_ values (Δ*C*_t_ *Ty‐1 (C*_t_ *Ty‐1* – *C*_t_ *EF1α)* − Δ*C*_t_ MM *(C*_t_ *Ty‐1* – *C*_t_ *EF1α*)) are depicted, which represent *Ty‐1* expression relative to the level of the susceptible *ty‐1* allele in untransformed MM plants. Asterisks indicate significant differences between T_3_ and untransformed MM plants according to one‐way analysis of variance (***P < 0.001). (B) Virus titre quantification in T_3_ progeny of *Ty‐1* transformants. Values were normalized relative to *EF1α* and calibrated to the levels in untransformed MM plants, which were set to 1. Asterisks indicate significant differences between T_3_ and untransformed MM plants according to one‐way analysis of variance (***P < 0.001). (C) Symptoms on infection with mock‐inoculation, TYLCV and BCTV in MM and in different individuals of two T_4_ families of *Ty‐1*‐transformed tomato plants at 50 days post‐inoculation (dpi). In the upper left corners, the disease symptom scores are depicted. (D) Virus titre quantification in plants from two T_4_ families (35S::*Ty‐1*), a *Ty‐1* introgression line and untransformed MM. Values are calibrated to tomato *EF1α* and the fold difference was calculated compared to (susceptible) MM plants inoculated with TYLCV or BCTV (set to 1). Asterisks represent significant differences to the TYLCV‐ or BCTV‐challenged MM samples according to one‐way analysis of variance (***P < 0.001).

To rule out any other chromosomal introgression(s) being involved, and to ensure that the broad‐spectrum resistance was solely due to the expression of the *Ty‐1* gene, susceptible tomato MM was transformed with a copy of the *Ty‐1* gene for a functional complementation study. *Agrobacterium*‐mediated transformation of MM plants with a 35S promoter‐driven *Ty‐1* construct resulted in nine primary transformants (T_1_). After selfing of the T_1_ plants, a T_2_ progeny was obtained that was challenged with TYLCV and monitored for phenotypic responses. In all the subsequent experiments, the Almeria isolate of TYLCV was used as representative of the *Begomovirus* genus, as was also done in previous *Ty‐1* research (Butterbach *et al.*, 2014; Verlaan *et al.*, 2013). Several T_2_ families showed a clear segregation for TYLCV resistance (mild symptoms), correlated to the presence of the *Ty‐1* transgene, as scored by a positive PCR amplification of the *NPTII* gene cassette and *Ty‐1* insertion (data not shown). T_2_ plants showing resistance to TYLCV were used for selfing to produce a T_3_ generation. After another round of TYLCV challenges, two T_3_ families were selected from which all seedlings exhibited TYLCV resistance. These two families originated from two different primary transformants. A reverse transcription (RT)‐qPCR analysis on samples collected from these two families revealed a significant increase in the expression level of *Ty‐1*, which may vary between lines depending on integration site or number (Fig. 2A). The relative fold difference was calculated via the 2^−ΔΔ*C*t^ method, showing a 20‐ to 30‐fold increase in *Ty‐1* expression levels in *Ty‐1*‐transformed lines relative to the *ty‐1* allele (detected by the same primer set) from susceptible MM plants. Furthermore, a *c*. 10‐fold reduction was observed in TYLCV titres in the transgenic lines relative to the untransformed, susceptible MM plants (Fig. 2B). T_3_ individuals from these two lines that exhibited significantly higher *Ty‐1* expression levels and reduced virus accumulation were selected and selfed to produce two homozygous T_4_ lines for further analysis (T_4_ lines 1 and 2).

To complement and support the findings on the resistance spectrum of *Ty‐1*, batches of ten seedlings from the stable *Ty‐1* transformants (T_4_ line 1 and 2) were challenged with TYLCV and BCTV, and monitored for their phenotypic responses. All seedlings from both lines showed resistance against TYLCV and displayed no disease symptoms (Fig. 2C). However, upon a challenge with BCTV, one out of ten plants from T_4_ line 1 and four out of ten from T_4_ line 2 ended up showing mild BCTV symptoms (Fig. 2C). Systemic leaves were collected at 50 days post‐inoculation (dpi) and viral titres of both TYLCV and BCTV were determined by qPCR in both lines and compared to the titres in MM and the *Ty‐1* tomato introgression line. The results showed comparable levels of viral DNA accumulation for TYLCV and BCTV in both the *Ty‐1* introgression line and *Ty‐1* transgenic lines, and clearly reduced relative to the titres obtained from susceptible MM. However, the reduction in BCTV accumulation in both T_4_ lines was less profound than that of TYLCV (Fig. 2D). Plants from the two T_4_ lines that exhibited mild BCTV symptoms showed relatively higher BCTV titres compared to plants that remained symptomless.

### Transgenic expression of *Ty‐1* in *N. benthamiana* confers resistance to TYLCV and BCTV

**Figure 3:**
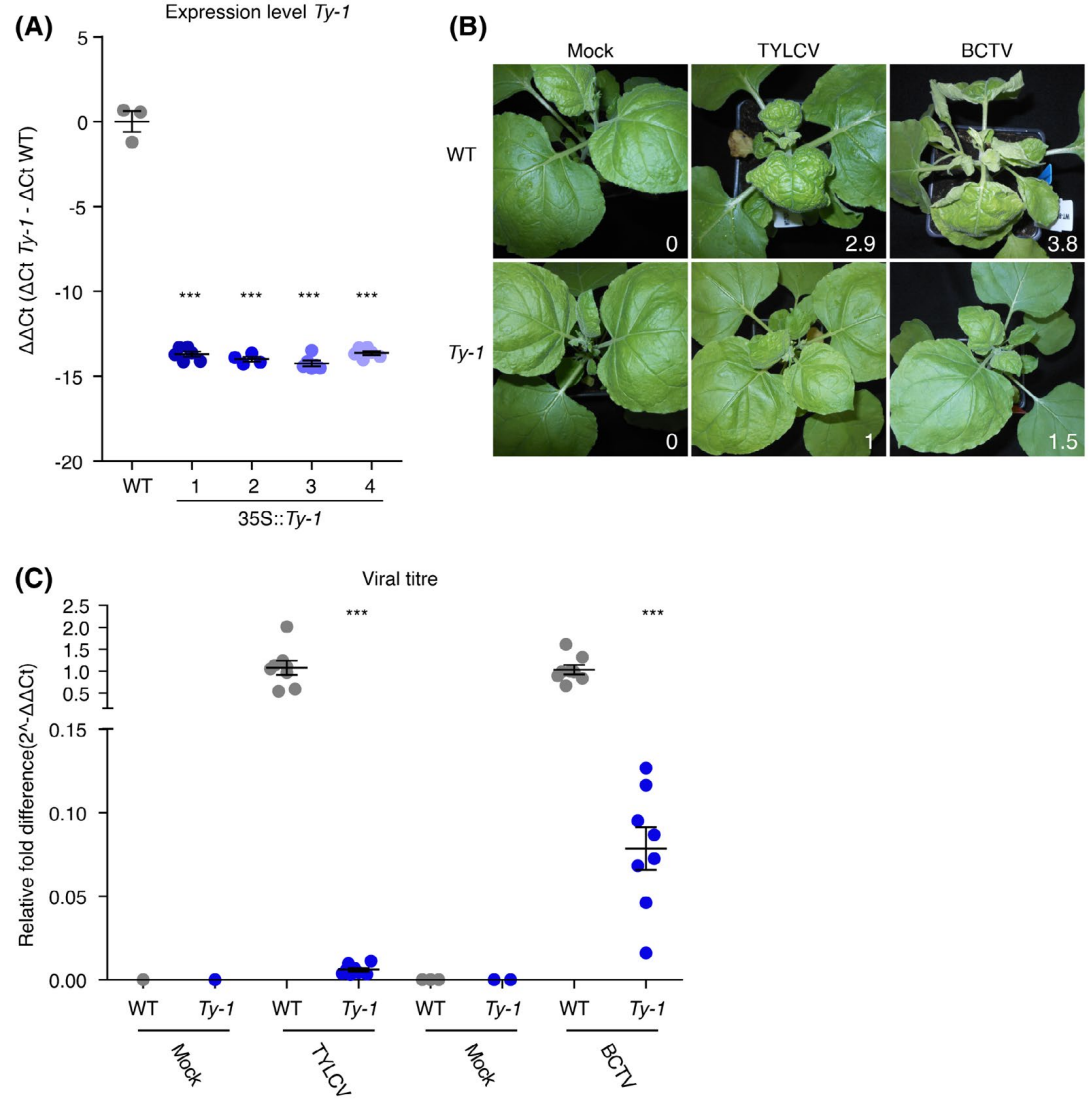
*Ty‐1* transgenic *Nicotiana benthamiana* plants are resistant to TYLCV and BCTV. In all graphs, every point represents one plant and lines represent means and standard error of the mean of biological replicates. (A) Four independent transformant lines were generated and the expression level of *Ty‐1* was measured. Values were normalized relative to *EF1α*. The ΔΔ*C*_t_ values (Δ*C*_t_ *Ty‐1 (C*_t_ *Ty‐1* – *C*_t_ EF*1α)* − Δ*C*_t_ WT *(*C_t_ *Ty‐1* – *C*_t_ *EF1α*)) are depicted, which represent *Ty‐1* expression relative to wild‐type (WT) plants. Asterisks indicate significant differences between WT plants and *Ty‐1* transformations according to one‐way analysis of variance (***P < 0.001). (B) Symptoms upon infection with mock‐inoculation, TYLCV or BCTV in WT and *Ty‐1* transgenic *N. benthamiana* plants. Pictures were taken at 18 days post‐inoculation (dpi) and the symptom scores are depicted in the lower right corners. (C) Virus titre quantification in WT and *Ty‐1 t*ransgenic *N. benthamiana* plants upon challenge with BCTV or TYLCV. 18 days post‐BCTV/TYLCV infection, young uninoculated leaves were harvested. Total DNA was isolated and viral titres were measured compared to the presence of genomic DNA with primers amplifying the gene for 25S rRNA. The relative fold difference is depicted, which is calculated via the 2^−ΔΔ*C*t^ method (WT infected plants set to 1). Note that the *y*‐axis is split to show the low titres. Asterisks indicate significant differences between WT plants and *Ty‐1* transformants according to one‐way analysis of variance (***P < 0.001).

To further substantiate the findings on broad‐spectrum resistance conferred by *Ty‐1*, and to test whether the *Ty‐1* resistance gene also functions in other Solanaceae species, stable transformants of *N. benthamiana* plants were generated expressing *Ty‐1* driven by a 35S promoter. In analogy to the transformation of tomato MM (previous section), stable transformants were selected based on a TYLCV resistant phenotype and selfed until T_4_. Expression levels of *Ty‐1* were determined by RT‐qPCR in four transgenic lines selected from two independent transformations (Fig. 3A). The four transgenic lines showed a comparable high level of expression of *Ty‐1*. Whereas values for *C*_t_ were in the range of *c*. 25 for all four transgenic lines, no signal was obtained from wild‐type (WT) *N. benthamiana* samples due to the absence of a *ty‐1* homologue in WT *N. benthamiana* that could be amplified by the primers used. The *C*_t_ values were normalized to the expression of the housekeeping gene Elongation Factor *1α* (*EF1α*), and the ΔΔ*C*_t_ values were calculated to compare *Ty‐1* transgenic plants with WT *N. benthamiana*, showing clear and high expression levels in *Ty‐1* plants. On infection with TYLCV no clear symptoms were observed in all four lines (Fig. 3B), and when virus titres were determined a *c*. 100‐fold reduction was observed in the transgenic lines compared to WT *N. benthamiana* plants (Fig. 3C).

When the stable *Ty‐1 N. benthamiana* transformants were challenged with BCTV, only a slight curling and chlorosis (average symptom score 1.5) was observed at 18 dpi, whereas WT *N. benthamiana* revealed a strong curling and major chlorosis of systemic leaves (average symptom score 3.8) (Fig. 3B). BCTV titres showed a *c*. 15‐fold reduction relative to the titres obtained from susceptible *N. benthamiana*, which was smaller than the difference in TYLCV titres (between 70‐ and 180‐fold) (Fig. 3C). This smaller effect on BCTV compared to TYLCV was consistently observed during repeated experiments and resembled the observations in the *Ty‐1* tomato introgression line and transgenic tomato.

### Co‐replication of a betasatellite compromises resistance by *Ty‐1*

**Figure 4:**
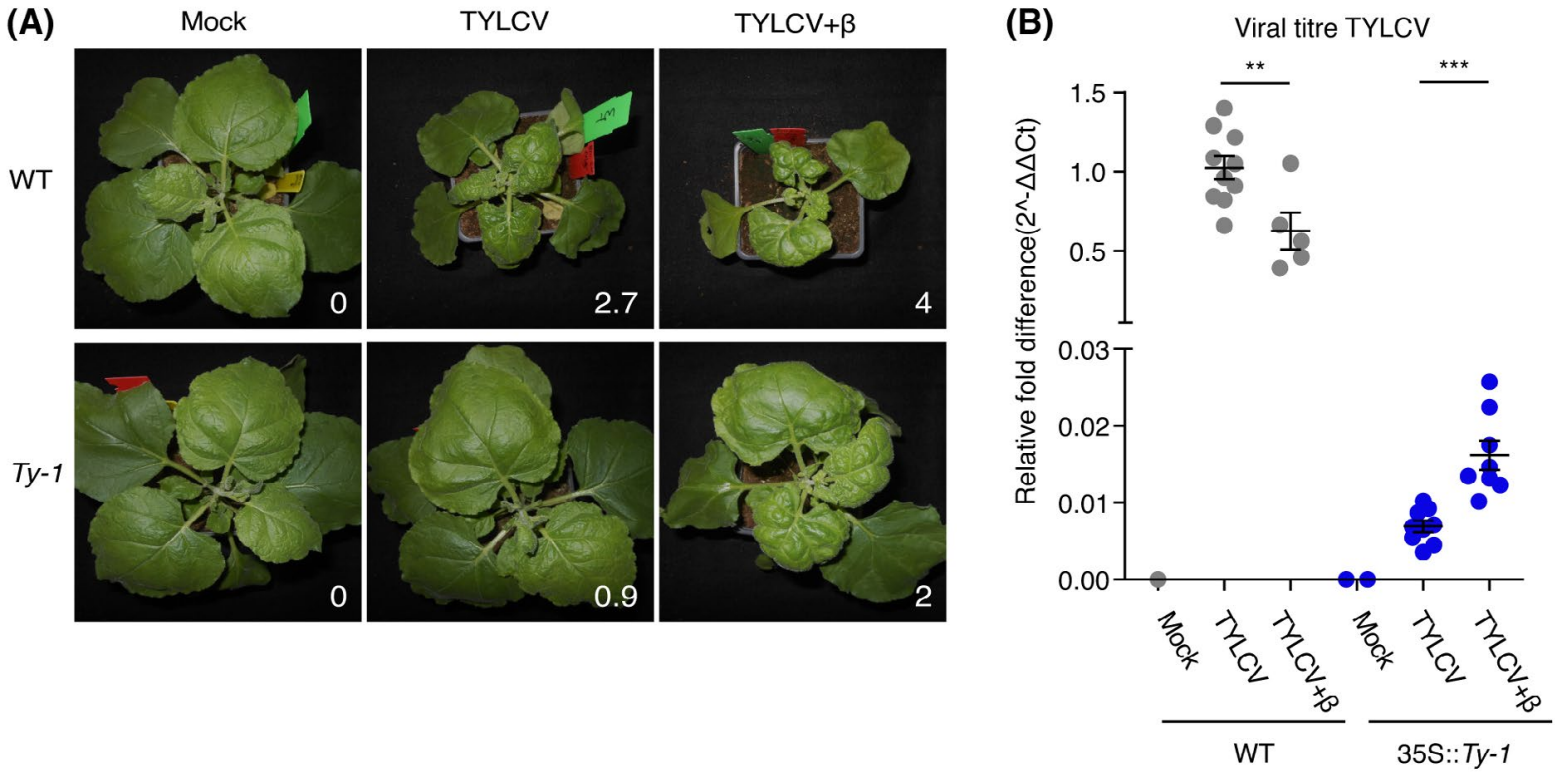
Breaking of resistance by co‐replication of AYVB in *Ty‐1* transgenic *Nicotiana benthamiana*. (A) Pictures taken 19 days post‐inoculation (dpi) from wild‐type (WT) and 35S::*Ty‐1* plants infected with TYLCV only or co‐infected with AYVB (denoted as β in the figure). The symptom score is indicated at the bottom right corner. (B) Systemically infected leaves of WT and 35S::*Ty‐1 N. benthamiana* plants were harvested 19 dpi and DNA was isolated. The viral titre was determined via quantitative PCR relative to the presence of genomic DNA (25S rRNA) and the relative fold difference was calculated via the 2^−ΔΔ*C*t^ method and normalized to the group WT infected with TYLCV (set to 1). Every point represents one plant. Lines represent means and standard error of the mean of biological replicates. Note that the *y*‐axis is split to show the low titres. Asterisks indicate significant differences between plants infected with TYLCV only or co‐infected with AYVB according to *t*‐test analysis (**P < 0.01; ***P < 0.001).

Earlier, a co‐infection of CMV was shown to compromise *Ty‐1* resistance (Butterbach *et al.*, 2014), likely due to the abrogation of AGO4 activity by the CMV 2b RNAi suppressor protein. Under natural field conditions TYLCV is often observed with co‐replicating betasatellites, elements that encode suppressors of TGS (Yang *et al.*, 2011). To test whether betasatellites also compromise *Ty‐1* resistance, susceptible MM and *Ty‐1* introgression tomato plants were co‐infected with TYLCV and an ageratum yellow vein virus associated betasatellite (AYVB) via agroinfiltration. In parallel, WT *N. benthamiana* plants were infected with either TYLCV alone or in combination with the betasatellite. At 21 dpi, no differences in viral symptoms were observed between TYLCV singly infected and TYLCV plus betasatellite co‐infected tomato plants (results not shown). However, PCR analysis of systemically infected tomato leaves for the presence of the betasatellite turned out negative (data not shown), indicating that the betasatellite did not co‐replicate with TYLCV in tomato. In contrast to tomato, at 19 dpi curling and yellowing of systemically infected leaves was more severe in *N. benthamiana* plants co‐infected with TYLCV and the betasatellite compared to plants only infected with TYLCV (Fig. 4A). The presence of the betasatellite in systemically infected leaves was confirmed by PCR (data not shown). For this reason, the compromising nature of betasatellites, and their encoded TGS suppressor protein, on *Ty‐1* resistance was further analysed in stably transformed *N. benthamiana Ty‐1* plants. To this end, *Ty‐1* stable transformants, next to WT *N. benthamiana* plants, were challenged with TYLCV singly or in a mixed setting with the betasatellite. At 19 dpi WT plants again showed yellowing and curling of the top leaves in the presence of TYLCV, whereas the transgenic *Ty‐1 N. benthamiana* plants only exhibited very mild symptoms. In the additional presence of the betasatellite both WT and transgenic plants exhibited more severe yellowing and curling, although the symptoms in the transgenic plants infected with TYLCV and the betasatellite were less pronounced than in WT plants infected with TYLCV only (average symptom score WT + TYLCV of 2.7, *Ty‐1* + TYLCV + betasatellite of 2, see Fig. 4A). When virus titres were determined by qPCR, a co‐infection with the betasatellite led to a *c*. 2‐fold increase of TYLCV titres in systemically infected leaves from *Ty‐1* transgenic plants, while a reduction in virus titres was observed in WT (susceptible) plants (Fig. 4B). This difference was observed in all four transgenic lines and during two independent repetitions.

### Co‐expression of βC1 is sufficient to compromise *Ty‐1* resistance

**Figure 5:**
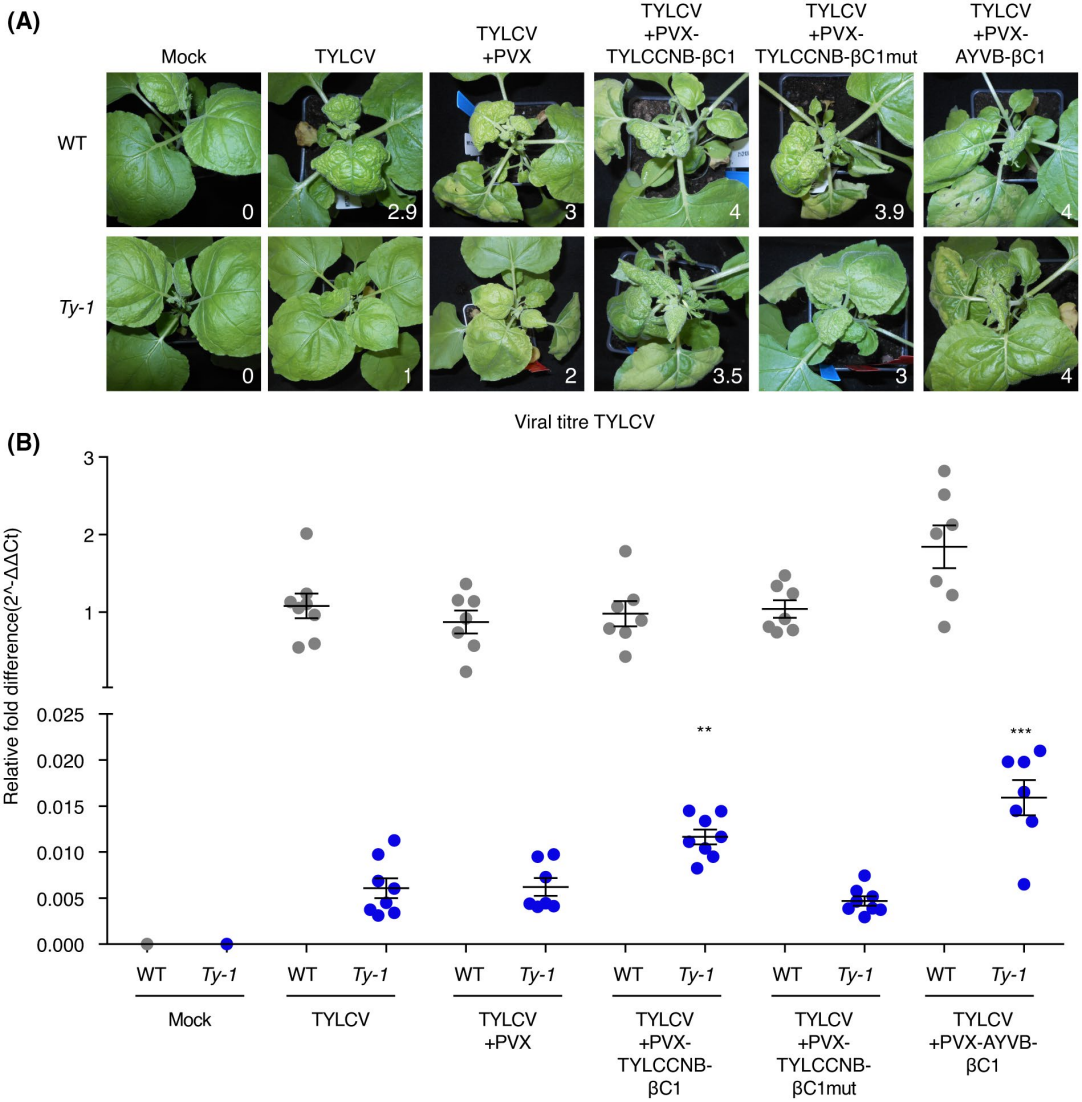
βC1 protein expressed via a PVX vector compromises resistance against TYLCV in *Ty‐1* transgenic *Nicotiana benthamiana*. (A) Wild‐type (WT) and *Ty‐1* transgenic *N. benthamiana* plants were infected with TYLCV and at 10 days post‐inoculation (dpi) were challenged with PVX expressing TYLCCNB‐βC1, TYLCCNB‐βC1 mutant, AYVB‐ βC1 or empty PVX vector. Eight days post‐PVX infection, pictures were taken and plants were scored for disease symptoms, indicated in the bottom right corner. (B) From the plants described under 5a, samples of systemically infected leaves were collected, DNA was isolated and the titre of TYLCV was determined via quantitative PCR. The viral titre was normalized to the presence of genomic DNA (25S rRNA) and the fold difference was calculated according to the 2^−ΔΔ*C*t^ method relative to WT plant infected with TYLCV only (set to 1). Every point represents one plant. Lines represent means and standard error of the mean of biological replicates. Note that the *y*‐axis is split to show the low titres. Asterisks indicate significant differences according to one‐way analysis of variance. In the analysis WT TYLCV‐only infected plants were compared with the different WT co‐infected groups and the same was done for the *Ty‐1* transformants (**P < 0.01; ***P < 0.001).

To test whether the compromising effect of the betasatellite on *Ty‐1* resistance against TYLCV was due to co‐replication of the betasatellite with TYLCV or to suppression of TGS by the betasatellite encoded protein βC1, experiments were repeated, but this time *Ty‐1* stable transformants were challenged with TYLCV in the presence of a potato virus X (PVX) vector expressing the βC1 protein. Considering that so far βC1 protein TGS suppression activity has been reported only for tomato yellow leaf curl China virus associated betasatellite (TYLCCNB) and in the experiments here AYVB was used, TYLCCNB‐βC1 and a functionally deficient mutant were included as positive and negative control, respectively (Cui *et al*., 2005; Yang *et al*., 2011). Twenty‐five‐day‐old WT and *Ty‐1* transgenic *N. benthamiana* plants were infected with TYLCV via agroinfiltration and at 10 dpi were challenged with PVX from which no protein, the βC1 protein of AYVB, the βC1 protein of TYLCCNB or the mutated TYLCCNB‐βC1 protein was expressed. The challenge with PVX was optimized at 10 days post‐TYLCV agroinfection due to PVX causing systemic infections much faster than TYLCV. Eight days post‐PVX infection plants were analysed for development of disease symptoms caused by TYLCV and/or PVX. Since PVX (empty vector) infections also caused chlorosis of the top leaves, these plants were more difficult to score for TYLCV symptoms. Whereas WT *N. benthamiana* plants infected with TYLCV showed symptoms as earlier observed (Fig. 5A), a mixed infection with PVX‐TYLCCNB‐βC1 or PVX‐AYVB‐βC1 intensified the curling and chlorosis (Fig. 5A) similarly to that during a co‐infection with the betasatellite (Fig. 4). During a co‐infection of TYLCV and the PVX‐TYLCCNB‐βC1 mutant (encoding a nonfunctional mutant βC1 TGS suppressor) only some necrosis and increased chlorosis was observed compared to TYLCV only. In the stably transformed *Ty‐1 N. benthamiana* plants, TYLCV only caused some slight chlorosis, but the additional presence of PVX‐AYVB‐βC1 or PVX‐TYLCCNB‐βC1, and not of PVX‐TYLCCNB‐βC1mutant, caused severe curling and chlorosis. To determine whether the increase in symptoms in the susceptible WT and *Ty‐1* transgenic *N. benthamiana* plants also correlated with a higher TYLCV titre, samples were collected from systemically infected leaves and DNA purified for qPCR analysis. In WT *N. benthamiana* no significant change in virus titres was observed with TYLCV only versus a mixed infection with TYLCV and one of the PVX constructs (Fig. 5B). In the case of a mixed infection with PVX‐AYVB‐βC1 a trend of increased TYLCV titres was observed, which contrasted with the reduced titres seen in the presence of AYVB. In the *Ty‐1* transgenic plants challenged with TYLCV, virus titres were again drastically reduced compared to WT plants, and these titres did not change with the addition of PVX‐empty or PVX‐TYLCCNB‐βC1 mutant. However, in the additional presence of PVX‐AYVB‐βC1 or PVX‐TYLCCNB‐βC1 TYLCV titres increased 2.7‐ and 2‐fold, respectively (Fig. 5B). These data indicate that the presence of a betasatellite compromises *Ty‐1* resistance by means of its encoded TGS suppressor, the βC1 protein.

## Discussion

Earlier we showed that *Ty‐1* confers resistance against the monopartite TYLCV (Israel strain, Almeria isolate) and the bipartite begomovirus ToSRV. We also showed that *Ty‐1* resistance involves an amplification of the antiviral RNAi response, leading to increased levels of viral siRNA production and concomitant cytosine methylation within the viral DNA genome (Butterbach *et al.*, 2014). Here it is shown that, besides TYLCV, *Ty‐1* also confers resistance to TYLCSV, another species from the TYLCV cluster of begomoviruses, and to the completely distinct leafhopper‐transmitted curtovirus BCTV. Furthermore, stable transformants of susceptible tomato MM and *N. benthamiana* expressing a *Ty‐1* transgene exhibited the same resistance spectrum as tomato *Ty‐1* introgression lines, excluding the involvement of other resistance loci in this broad‐spectrum resistance. In the presence of a betasatellite, but also on trans‐complementation of the βC1 protein expressed from a PVX virus vector, *Ty‐1* resistance against TYLCV was compromised. Altogether, these results demonstrate that *Ty‐1* provides resistance to a wide range of geminiviruses, which is not restricted to begomoviruses only, and is compromised by suppressors of RNAi that interfere at TGS.

Whereas this study shows for the first time that *Ty‐1* also hampers the replication of a curtovirus, the findings on the effectiveness of *Ty‐1* against a group of TYLCV‐like viruses are supported and in agreement with the outcome of other studies. Others have reported on the efficacy of *Ty‐1* against the monopartite TYLCV‐Mld (Shahid *et al.*, 2013), TYLCSV (Barbieri *et al.*, 2010), tomato curly stunt virus (ToCSV), tomato leaf curl Bangalore virus (ToLCBV), honeysuckle yellow vein mosaic virus (HYVMV) and tobacco leaf curl Japan virus (TbLCJV) (Pietersen and Smith, 2002; Prasanna *et al.*, 2015; Shahid *et al.*, 2013) and to multiple bipartite begomoviruses including tomato mottle virus (ToMoV), tomato leaf curl New Delhi virus (ToLCNDV) and tomato leaf curl Palampur virus (ToLCPalV) (Prasanna *et al.*, 2015). In our earlier study, *Ty‐1* was shown to confer resistance to the bipartite begomovirus ToSRV as well (Butterbach *et al.*, 2014).

*Ty‐1* does not confer resistance to RNA viruses, as recently shown for tomato spotted wilt virus (TSWV) and CMV (Butterbach *et al.*, 2014). Also in the study presented here, the symptoms caused by a PVX infection were not different between WT and stably transformed *Ty‐1 N. benthamiana*. However, RNA viruses may compromise *Ty‐1* resistance against geminiviruses, as shown earlier during a co‐infection of TYLCV and CMV. The observed increase in TYLCV titres in the latter case was explained to be due to inhibition of the TGS response by the CMV 2b RNAi suppressor protein (Butterbach *et al.*, 2014; González *et al.*, 2010; Hamera *et al.*, 2012). This idea is strengthened by the current study, showing a compromising effect on *Ty‐1* resistance by AYVB, and also solely by the AYVB βC1 and TYLCCNB βC1 proteins expressed from a PVX virus vector. TYLCCNB βC1 protein has been demonstrated to suppress TGS via inhibition of the enzyme *S*‐adenosylhomocysteine hydrolase (SAHH), an enzyme that is required for production of the methyl donor used in the TGS pathway (Yang *et al.*, 2011). CMV 2b in contrast has been shown to hamper TGS via inhibition of AGO4 (González *et al.*, 2010; Hamera *et al.*, 2012). Despite the fact that these viral suppressors act differently, the ability to inhibit TGS at (any) different step(s) of the pathway apparently is sufficient to compromise *Ty‐1*‐mediated resistance*.*

Recently, Conflon *et al. *(2018) reported on the compromising effect of a co‐replicating betasatellite on *Ty‐1* resistance. *Ty‐1* tomato lines co‐infected with different TYLCV strains and a cotton leaf curl Gezira betasatellite revealed an increase in disease symptoms, but interestingly titres only increased in the mixed infection case with TYLCV‐Il, while those of TYLCV‐Mld were decreased on addition of the betasatellite. How to explain these effects caused by one and the same betasatellite remains to be further investigated, although it is likely that this has to do with the virus strains and not the betasatellite. Considering that the efficacy of *Ty‐1* is compromised by trans‐complementing TGS suppressors, the suppressors encoded by the geminiviral DNA genome of TYLCV‐Il and TYLCV‐Mld will also affect *Ty‐1* resistance. If their suppressors of RNAi differ in strength, *Ty‐1*‐mediated TGS will be suppressed to varying degrees, leading to different levels of reduction in virus titres. Although speculative, whether this also explains the observed difference in efficacy of *Ty‐1* resistance towards TYLCV and BCTV remains to be analysed. To this end, initial attempts to detect TGS suppression by BCTV and TYLCV in 16C‐TGS transgenic plants (as shown by Buchmann *et al.*, 2009) failed and remain to be repeated.

Recently several articles have been published on *Ty‐1* resistance‐breaking strains of TYLCV in cultivations of *Ty‐1*‐bearing tomato. Samples of *Ty‐1* plants showing TYLCV‐like symptoms collected in Morocco, Italy and Spain revealed the presence of viruses derived from a recombination event between TYLCV and TYLCSV in which a noncoding region between the origin of replication and the start of the coding region of V2 were exchanged (Belabess *et al.*, 2015; Granier *et al.*, 2019; Panno *et al.*, 2018; Torre *et al.*, 2019). In Morocco, this recombinant replaced both parental strains, but also under laboratory conditions this recombinant was positively selected in *Ty‐1*‐bearing plants (Belabess *et al.*, 2015, 2016). So far, the mechanism behind the improved fitness of this resistance‐breaking strain is unknown, but the region of recombination covers the promoter region of the coat protein. The resistance breaking, therefore, might be due to (a combination of) changes in replication efficiency or viral gene expression, or the region of recombination might be less prone to TGS.

In conclusion, *Ty‐1* is a unique resistance gene that confers resistance to a broad spectrum of geminiviruses, as demonstrated for begomoviruses and a curtovirus. Although *Ty‐1* is likely to confer resistance to all geminiviruses, suppression of RNAi by co‐replicating betasatellites, or other RNA viruses, but also by suppressors encoded from the geminiviral DNA genome itself, seems to present the Achilles’ heel of the resistance mechanism. Whether *Ty‐1* resistance can also be compromised by an infection involving a bacterial or fungal/oomycete pathogen, some of which are shown to either cause hypomethylation or suppress (the amplification of) RNAi (Hou *et al.*, 2019; Navarro *et al.*, 2008; Pavet *et al.*, 2006), remains an interesting question. The effect of co‐infections on *Ty‐1* resistance is important in light of disease management strategies, and simultaneously underlines the importance for monitoring the presence of other pathogens that encode suppressors of RNAi and interfere at the level of TGS during cultivation of *Ty‐1*‐bearing tomato plants.

## Experimental Procedures

### Plant material and virus sources

Throughout this study *S. lycopersicum* and *N. benthamiana* plants were maintained under greenhouse conditions at 23 °C during the day and at 21 °C at night (16 h light/8 h dark regime) and at a relative humidity of 60%. Tomato cv. Moneymaker (MM) was used as susceptible control and a *Ty‐1* introgression line was derived from *S. chilense* LA1969 (Verlaan *et al.*, 2013). Plants were infected with geminiviruses by means of agroinoculation of infectious clones. Agroinfectious clones used in this study were from two isolates of TYLCV, namely the Israel strain isolated from Almeria, Spain, as described by Morilla *et al. *(2005) (GenBank AJ489258.1), and the Israel strain isolated from Shanghai, China, named TYLCV‐[CN:SH2] [GenBank AM282874, (Zhang *et al.*, 2009)], an isolate of TYLCSV [GenBank X61153.1 (Noris *et al.*, 1998)] and an isolate of BCTV California Logan [GenBank M24597.2 (Stanley *et al.*, 1986)]. Co‐infections were performed with an agroinfectious clone of AYVB [GenBank AJ252072.1 (Saunders *et al.*, 2000)].

### Transformation of tomato and *N. benthamiana* plants with *Ty‐1*

To generate a construct for transformation purposes, the *Ty‐1* full‐length coding sequence was amplified from complementary DNA (cDNA) of *Ty‐1* introgression lines with forward primer Ty‐1‐CDS‐F and reverse primer Ty‐1‐CDS‐R (for primer sequences see Table S1) (Verlaan *et al.*, 2013). PCR products were cloned into the pENTR/D‐TOPO vector (Invitrogen, Carlsbad, CA, USA) and verified by sequence analysis prior to recombination into pK7WG2 via an LR reaction (Invitrogen). The resulting plasmid was transferred to *Agrobacterium tumefaciens* strain AGL1 by electroporation. Transformation of cotyledons from MM and *N. benthamiana* was carried out according to the method described by Appiano *et al. *(2015). Plants regenerated from kanamycin‐resistant primary transformants (T_1_) were selected and self‐pollinated to produce T_2_ families. From each segregating T_2_ family, transgenic lines were selected by PCR amplification of the *NPTII* gene cassette [using primers NPT3 and NPT4 (Heilersig *et al.*, 2006), amplifying a 722‐bp fragment] and a partial *Ty‐1* gene sequence (using primers CaMV 35S promoter primer 35S‐F and gene insert‐specific primer Ty‐1‐R, amplifying a 822‐bp fragment).

### PVX constructs

PVX infectious clones encoding the betasatellite C1 protein gene were constructed as follows. The open reading frame of AYVB C1 was amplified from the infectious clone (GenBank AJ252072.1) using primers AYVB‐C1‐F and AYVB‐C1‐R (Table S1). Sequences for C1 and a C1 mutant (Cui *et al.*, 2005) of the TYLCCNB were ordered as a gene block from Integrated DNA Technologies (IDT, Leuven, Belgium) and were based on GenBank accession AJ421621.1. The gene blocks were cloned into pJet1.2 according to the manufacturers’ protocol (Thermo Fisher, Waltham, MA, USA), and their sequences verified. Next, the TYLCCNB C1 gene was amplified with primers TYLCCNB‐C1‐F and TYLCCNB‐C1‐R containing a 5’ *Cla*I or *Sal*I restriction site. Amplicons were purified and subsequently digested with *Sal*I and *Cla*I prior to cloning into the *Sal*I‐ and *Cla*I‐digested PVX vector pGR107 (Jones *et al.*, 1999). Positive clones were selected and verified by sequence analysis. The *Agrobacterium* strain GV3101, containing the helper plasmid pSoup, was transformed with the constructs PVX‐AYVB‐βC1, PVX‐TYLCCNB‐βC1, PVX‐TYLCCNB‐βC1mut or with the empty pGR107 plasmid for subsequent agroinfiltration of plants.

### *Agrobacterium* transient transformation assay

*Agrobacterium* transient transformation assays were performed following a slightly modified protocol of Bucher *et al. *(2003). In brief, *A. tumefaciens* was grown overnight at 28 °C in 3 mL LB3 (10 g/L tryptone, 5 g/L yeast, 4 g/L NaCl, 1 g/L KCl, 3 g/L MgSO_4_.2H_2_O) medium containing proper antibiotic selection pressure. From this culture, 600 μL was transferred and incubated overnight in 3 mL induction medium [10.5 g/L K_2_HPO_4_, 4.5 g/L KH_2_PO_4_, 1 g/L(NH_4_)_2_SO_4_, 0.5 g/L sodium citrate.2H_2_O, 1 mM MgSO_4_.7H_2_O, 0.2% (w/v) glucose, 0.5% (v/v) glycerol, 50 μM acetosyringone, 10 mM 2‐(*N*‐morpholino)ethanesulphonic acid (MES), pH 5.6]. The next day bacteria were pelleted by centrifugation (15 min, 2670 *g*) and resuspended in MS MES buffer [Murashige and Skoog medium (Duchefa Biochemie, Haarlem, Netherlands) supplemented with 150 μM acetosyringone, 10 mM MES and 87 mM sucrose] at an OD_600_ of 0.5 per construct. One hour after plants were watered in excess, the first two true leaves were fully infiltrated with this mixture by pressure inoculation with a needleless syringe on the abaxial side of the leaf.

### Disease assessment

Plant responses were scored several times during the whole disease assay on viral symptom development. The time span of the disease assay varied per experiment and lasted up to 50 days. Depending on the lines and experiments, five to ten plants were challenged. Each plant was rated using a 0 to 4 disease severity index (DSI) described by Friedmann *et al. *(1998), where 0 indicates no viral disease symptoms and 4 means severe symptoms. Intermediate scores (0.5, 1.5, 2.5 and 3.5) were incorporated for more precise scoring.

### Nucleic acid purification

Systemically infected leaves were harvested and snap frozen in liquid nitrogen. The samples were ground either by mortar and pestle or by using precellys (Bertin Instruments, Montigny‐le‐Bretonneux, France) for 10 s at 5000 rpm. To determine geminivirus titres, DNA was isolated following the cetyltrimethyl ammonium bromide (CTAB) method of Doyle and Doyle (1987) with slight modifications as described by Fulton *et al. *(1995). In brief, ground plant material was incubated for 1 h at 65 °C with CTAB buffer (0.1 M Tris, 0.7 M NaCl, 0.01 M EDTA, 2% CTAB). After chloroform/isoamyl alcohol extraction and spinning of the samples, the upper aqueous phase was transferred to a new tube and the DNA precipitated from the suspension by adding isopropanol in a 1:1 ratio. DNA was pelleted by centrifugation for 10 min at 9000 *g*, dried and dissolved in Milli‐Q water. For *Ty‐1* expression analysis, RNA from *N. benthamiana* plants was extracted using TRIzol according to the manufacturers’ protocol (Invitrogen) and RNA from tomato plants by using the RNeasy Plant Mini Kit (Qiagen, Hilden, Germany) following the manufacturer’s protocol. DNA and RNA concentrations were measured with a NanoDrop ND‐1000 device.

### Detection of the betasatellite

DNA of AYVB was amplified using AYVB‐specific primers AYVB‐F1, AYVB‐F2 and AYVB‐R (Table S1). The combination of AYVB‐F1 and AYVB‐R resulted in a product of 823 bp, whereas AYVB‐F2 and AYVB‐R resulted in a product of 457 bp. The PCR was performed with GoTaq polymerase according to the manufacturers’ protocol (Promega, Madison, WI, USA), using the following cycling conditions: 5 min at 95 °C; 40 cycles of 30 s at 95 °C, 30 s at 55 °C, 60 s at 72 °C; 7 min at 72 °C. Amplified PCR products were visualized in a 1% agarose gel using ethidium bromide.

### Virus titration

Relative virus titres of the TYLCV Almeria isolate, TYLCSV, TYLCV‐[CN:SH2] and BCTV were determined by qPCR using 25S rRNA (*N. benthamiana*) or *EF1α* (*S. lycopersicum,* Solyc06g005060) as internal control. To measure samples of *N. benthamiana*, the reaction mixture contained 1× Sybr Select (Applied Biosystems, Foster City, CA, USA), 375 nM forward primer, 375 nM reverse primer and 10 ng genomic DNA, while for *S. lycopersicum* samples the iQ SYBR Green supermix (Bio‐Rad, Hercules, CA, USA) was used. For PCR amplification the following primers were used (Table S1): for TYLCV Almeria isolate primers TYLCV‐Alm‐qPCR‐F and TYLCV‐Alm‐qPCR‐R (Powell *et al.*, 2012), for TYLCSV primers TYLCSV‐qPCR‐F and TYLCSV‐qPCR‐R, for TYLCV‐[CN:SH2] primers TYLCV‐CN‐qPCR‐F and TYLCV‐CN‐qPCR‐R, for BCTV either primers BCTV‐qPCR‐F1 and BCTV‐qPCR‐R1 or BCTV‐qPCR‐F2 and BCTV‐qPCR‐R2. During qPCR 25S rRNA was amplified using primers 25S‐qPCR‐F and 25S‐qPCR‐R, and *EF1α* using primers SlEF1a‐qPCR‐F and SlEF1a‐qPCR‐R.

The qPCR was performed in a Bio‐Rad CFX384 using the following cycling conditions: 2 min at 95 °C, 40 cycles of 15 s at 95 °C and 1 min at 60 °C, followed by a melting curve with 0.5 °C steps from 60 to 95 °C to determine PCR specificity. Relative viral titres were calculated using the ΔΔ*C*_t_ method (Livak and Schmittgen, 2001). Values were normalized relative to the internal control 25S rRNA or *EF1α*, and calibrated to levels of the control plants, which were set as 1.

### *Ty‐1* gene expression analysis

Prior to gene expression analysis 1 µg of purified total RNA of *N. benthamiana* samples was treated with TURBO DNase (Invitrogen) following the manufacturer’s instructions. First‐strand cDNA was synthesized using random hexamers (Roche, Basel, Switzerland) and M‐MLV reverse transcriptase according to the manufacturers’ protocol (Promega, Madison, WI, USA). The RT‐qPCR was performed in a total volume of 10 µL, containing 1 µL of 5× diluted cDNA, 1× Sybr Select (Applied Biosystems), 375 nM forward primer and 375 nM reverse primer. Total RNA of *S. lycopersicum* was treated with DNase I, Amplification Grade (Invitrogen) following the manufacturer’s instructions. cDNA was synthesized using the iScript cDNA Synthesis Kit (Bio‐Rad) and RT‐qPCR performed with the iQ SYBR Green supermix (Bio‐Rad). To determine *Ty‐1* expression levels, either Ty‐1‐qPCR‐F1 and Ty‐1‐qPCR‐R1 or Ty‐1‐qPCR‐F2 and Ty‐1‐qPCR‐R2 primers were used. The gene expression was measured relative to the housekeeping gene *EF1α* by using the primers nbEF1α‐qPCR‐F and nbEF1α‐qPCR‐R in the case of *N. benthamiana* samples or SlEF1a‐qPCR‐F and SlEF1a‐qPCR‐R in the case of *S. lycopersicum* samples (Table S1). RT‐qPCR was performed in a Bio‐Rad CFX384 using the same conditions as for the virus titration. Relative gene expression was calculated using the formula 2^−(Δ*C*_t_ *Ty‐1 (C*_t_ *Ty‐1* – *C*_t_ *EF1α)* − Δ*C*_t_ MM *(C*_t_ *Ty‐1* – *C*_t_ *EF1α*)) (Livak and Schmittgen, 2001).

## Conflicts of Interest

The authors declare no conflicts of interest.

## Supporting information


**Table S1** List of sequences of primers used in this research.
