# Supplementary material for: Ty‐1, a universal resistance gene against geminiviruses that is compromised by co‐replication of a betasatellite
Source: Mol Plant Pathol. 2019 Nov 22;21(2):160–72. doi: 10.1111/mpp.12885 (PMC6988424; doi:10.1111/mpp.12885)
Supplement: Supplementary file 1 — Table S1 List of sequences of primers used in this research. [file MPP-21-160-s001.docx]

| **Name** | **Sequence 5’-3’** |
| --- | --- |
| 25s-qPCR-F | ATAACCGCATCAGGTCTCCA |
| 25s-qPCR-R | CCGAAGTTACGGATCCATTT |
| 35S-F | GCTCCTACAAATGCCATCA |
| AYVB-C1-F* | CC**ATCGAT**ATGACTATATCATATACCAACG |
| AYVB-C1-R* | GC**GTCGAC**TTATACGGTTACATTCTTGTATAC |
| AYVB-F1 | AGTGCTGGTGACCTTGTTGAA |
| AYVB-F2 | TTCAGAAAAAATGGGAGCGCAGCG |
| AYVB-R | CGTGTCGCGAATTGATGCCG |
| BCTV-qPCR-F1 | GGATGGAAATGTGCTGACCT |
| BCTV-qPCR-F2 | GTTGGGTGCTGGTGGTATAG |
| BCTV-qPCR-R1 | CTACACGAAGATGGGCAACC |
| BCTV-qPCR-R2 | CGTTCTTCGCTCTCTGACTATC |
| GFP-qPCR-F | CCTGTCCTTTTACCAGACAACCA |
| GFP-qPCR-R | CCCAGCAGCTGTTACAAACTCA |
| nbEF1α-qPCR-F | AGCTTTACCTCCCAAGTCATC |
| nbEF1α-qPCR-R | AGAACGCCTGTCAATCTTGG |
| NPT3 | TCGGCTATGACTGGGCACAACAGA |
| NPT4 | AAGAAGGCGATAGAAGGCGATGCG |
| SlEF1a-qPCR-F | ATTGGAAACGGATATGCCCCT |
| SlEF1a-qPCR-R | TCCTTACCTGAACGCCTGTCA |
| Ty-1-CDS-F | CACCTTCAAGTATATACAGGAAAAATGGGTGATCCG |
| Ty-1-CDS-R | CTAGAGTATTTCCTGCAAAACCGATG |
| Ty-1-qPCR-F1 | CTGGGCGTGTTTTGGTCTAC |
| Ty-1-qPCR-F2 | GGCAAAATATGCAGCCAGGCTTTCC |
| Ty-1-qPCR-R1 | CTCAGTAGCAGCTGACCTCG |
| Ty-1-qPCR-R2 | TCAGTATGTATACGAGGTTCGCCGT |
| Ty-1-R | CTGAGGGCTTGCACAGGCCAAT |
| TYLCCNB-C1-F* | CC**ATCGAT**ATGTATCATCCACAACAAATAAACATG |
| TYLCCNB-C1-R* | GC**GTCGAC**TCATACATCTGAATTTGTAAATACATCATAC |
| TYLCSV-qPCR-F | ATGCTACGGTTGTTGGAGGT |
| TYLCSV-qPCR-R | TCGCCTGCTCTTGATGATTA |
| TYLCV-Alm-qPCR-F | TTCGTCTAGATATTCCCTATATGAGGAGGTA |
| TYLCV-Alm-qPCR-R | GGGAAGCCCATTCAAATTAAAGG |
| TYLCV-CN-qPCR-F | GCTCGTAGAGGGTGACGAAG |
| TYLCV-CN-qPCR-R | TCTGCAATCCAGGACCTACC |

*In bold the restriction site sequences of *SalI* and *ClaI* respectively.

**Supplemental table S1**: List of sequences of primers used in this research
